# Integrating genomics for chickpea improvement: achievements and opportunities

**DOI:** 10.1007/s00122-020-03584-2

**Published:** 2020-04-06

**Authors:** Manish Roorkiwal, Chellapilla Bharadwaj, Rutwik Barmukh, Girish P. Dixit, Mahendar Thudi, Pooran M. Gaur, Sushil K. Chaturvedi, Asnake Fikre, Aladdin Hamwieh, Shiv Kumar, Supriya Sachdeva, Chris O. Ojiewo, Bunyamin Tar’an, Nigusie Girma Wordofa, Narendra P. Singh, Kadambot H. M. Siddique, Rajeev K. Varshney

**Affiliations:** 1 Center of Excellence in Genomics and Systems Biology, International Crops Research Institute for the Semi-Arid Tropics (ICRISAT), Hyderabad, India; 2 The UWA Institute of Agriculture, The University of Western Australia, Perth, Australia; 3 ICAR—Indian Agricultural Research Institute (IARI), Delhi, India; 4 Department of Genetics, Osmania University, Hyderabad, India; 5 ICAR—Indian Institute of Pulses Research (IIPR), Kanpur, India; 6 Rani Lakshmi Bai Central Agricultural University, Jhansi, India; 7 International Crops Research Institute for the Semi-Arid Tropics (ICRISAT), Addis Ababa, Ethiopia; 8 International Center for Agriculture Research in the Dry Areas (ICARDA), Cairo, Egypt; 9 International Center for Agriculture Research in the Dry Areas (ICARDA), Rabat, Morocco; 10 International Crops Research Institute for the Semi-Arid Tropics (ICRISAT), Nairobi, Kenya; 11 Department of Plant Sciences, University of Saskatchewan, Saskatoon, Canada; 12 Ethiopian Institute of Agricultural Research (EIAR), Debre Zeit, Ethiopia

## Abstract

**Key message:**

Integration of genomic technologies with breeding efforts have been used in recent years for chickpea improvement. Modern breeding along with low cost genotyping platforms have potential to further accelerate chickpea improvement efforts.

**Abstract:**

The implementation of novel breeding technologies is expected to contribute substantial improvements in crop productivity. While conventional breeding methods have led to development of more than 200 improved chickpea varieties in the past, still there is ample scope to increase productivity. It is predicted that integration of modern genomic resources with conventional breeding efforts will help in the delivery of climate-resilient chickpea varieties in comparatively less time. Recent advances in genomics tools and technologies have facilitated the generation of large-scale sequencing and genotyping data sets in chickpea. Combined analysis of high-resolution phenotypic and genetic data is paving the way for identifying genes and biological pathways associated with breeding-related traits. Genomics technologies have been used to develop diagnostic markers for use in marker-assisted backcrossing programmes, which have yielded several molecular breeding products in chickpea. We anticipate that a sequence-based holistic breeding approach, including the integration of functional omics, parental selection, forward breeding and genome-wide selection, will bring a paradigm shift in development of superior chickpea varieties. There is a need to integrate the knowledge generated by modern genomics technologies with molecular breeding efforts to bridge the genome-to-phenome gap. Here, we review recent advances that have led to new possibilities for developing and screening breeding populations, and provide strategies for enhancing the selection efficiency and accelerating the rate of genetic gain in chickpea.

## Introduction

Chickpea (*Cicer arietinum* L.) is one of the most economically important food legume crops. It is grown on an estimated 14.56 Mio. hectares, producing 14.78 Mio. tons in more than 55 countries of the world (FAOSTAT 2017). Several biotic and abiotic stresses restrict chickpea productivity to ~ 1 t ha^–1^ despite having the potential to produce 3.5–4 t ha^–1^ under optimum growing conditions (Roorkiwal et al. 2018a). Chickpea productivity has steadily increased over the years since 1961, but its sensitivity to biotic and abiotic stresses has also increased, possibly due to use and reuse of limited number of germplasm accessions/donor parents (Muehlbauer and Sarker 2017). Drought and heat stresses can reduce chickpea yields by up to 70% (Varshney et al. 2014a). The crop is also sensitive to biotic stresses, such as dry root rot, collar rot, fusarium wilt, ascochyta blight, botrytis grey mould, Helicoverpa and seasonal weeds which further reduce yields (see Li et al. 2015). The challenges posed by various biotic and abiotic stresses must be addressed in order to enhance chickpea productivity.

A major challenge for crop breeders is increasing yields to feed the estimated 10 billion people globally by 2050 (Hickey et al. 2019). Breeding techniques have successfully developed superior crop varieties; however, the prolonged use of these approaches has failed to meet the yield and nutrition demands. For instance, the number of people facing micronutrient deficiency has surpassed 2 billion, and this is likely to increase in the coming years in countries with poor dietary diversity (Varshney et al. 2019a). Improved crop varieties with higher yields, improved nutrition and disease/pest resistance are therefore essential to meet increasing demands, particularly in South Asia and sub-Saharan Africa. Chickpea, an important source of dietary protein, carbohydrates, minerals and essential nutrients, has the potential to contribute to fighting malnutrition. The crop also enriches soil fertility by adding significant amounts of nitrogen (60–103 kg ha^–1^) to the soil through N_2_ fixation (Kurdali 1996).

Advances in next-generation sequencing (NGS) technology have considerably curtailed sequencing costs (see Varshney et al. 2009) resulting in evolution of genotyping methods from individual marker- to whole-genome sequencing-based genotyping. This has resulted in the development of large-scale genomic resources, including genome sequence assemblies, re-sequencing of few thousand lines, high-resolution genetic maps and a range of low- to high-density genotyping platforms. These genomic resources have been used to identify alleles and haplotypes associated with agronomic traits in chickpea (Varshney et al. 2019b). For instance, genotyping-by-sequencing and skim-sequencing-based bin mapping in chickpea resulted in fine mapping of the ‘*QTL-hotspot*’ region from ~ 7.74 Mb to ~ 300 kb, for drought tolerance-related traits (Jaganathan et al. 2015; Kale et al. 2015). In addition, the development of a high-throughput single-nucleotide polymorphism (SNP) genotyping platform ‘Axiom®*CicerSNP* Array’ has facilitated the construction of dense genetic maps to advance genetics and breeding efforts in chickpea (Roorkiwal et al. 2018a). Re-sequencing efforts using whole-genome re-sequencing (WGRS) have led to dissection of genetic diversity, population structure, domestication patterns, linkage disequilibrium and the unexploited genetic potential for chickpea improvement (Varshney et al. 2019a). Modern genomics technologies have the potential to speed up the process for trait mapping, gene discovery, marker development and molecular breeding, in addition to enhancing the rate of productivity gains in chickpea. Integration of genome-wide sequence information with precise phenotypic variation allows to capture accessions with low-frequency variants that may be responsible for essential phenotypes such as yield components, abiotic stress tolerance or disease resistance. Until now, chickpea breeding programmes have focused on improving yield and its component traits under biotic and abiotic stress conditions. However, the changes in climatic patterns have imposed challenges for enhancing yields, in addition to the growing demand to meet the health benefits of consumers. Therefore, there is a urgent need to develop superior climate-resilient chickpea varieties to address the nutritional issues of people from developing countries.

In this review, we highlight the technological advances that transformed chickpea from an orphan crop to a genomic resource enriched crop in the post-genomics era. We also discuss next-generation mapping populations and strategies for addressing the emerging constraints limiting chickpea productivity and update the recently released molecular breeding varieties of chickpea for commercial cultivation. We also propose a sequence-based holistic breeding approach that integrates genomics resources with breeding efforts to develop improved chickpea varieties with enhanced genetic gains.

## Genetic resources for trait discovery and utilisation

The diversity of genetic resources in crops is crucial for meeting the basic human food and nutritional demands and serves as an important asset for selection and crop improvement. Enormous germplasm wealth is stored in the global genebank, which has the key to further accelerate crop improvement efforts (McCouch et al. 2013). For chickpea, a global collection of about 100,000 accessions is maintained in 120 national and international genebanks in 64 countries (Upadhyaya et al. 2017). Of these, the International Crops Research Institute for the Semi-Arid Tropics (ICRISAT) genebank contains the largest share (20.8%), with 20,764 accessions from 59 countries followed by ICAR-National Bureau of Plant Genetic Resources (ICAR-NBPGR; 16%) and the International Center for Agricultural Research in the Dry Areas (ICARDA; 15%) representing more than 50% of the global collection of chickpea. The genetic resources conserved at the ICRISAT and ICARDA genebanks have been characterised for basic traits, with germplasm subsets developed for breeding purposes. These subsets, including core collection (1956 accessions), mini-core collection (211 accessions), global composite collection (3000 accessions), trait-based FIGS (Focused Identification of Germplasm Strategy) sets and reference set (300 accessions), are considered perfect resources for mining allelic diversity, dissecting population structure and association mapping in chickpea (Upadhyaya and Ortiz 2001; Upadhyaya et al. 2006, 2008). This will help to detect QTLs that can be deployed in breeding programmes to develop improved chickpea varieties.

Over the past decades, the narrow genetic base in breeding efforts, prolonged breeding cycles, delays in the adoption of modern technologies and partial seed delivery systems have limited the delivery of genetic gains in farmers’ fields (Varshney et al. 2018). Enhancing genetic diversity is a prerequisite to addressing the persistent challenges of quantitative trait dissection. For instance, several QTLs have been mapped for traits related to morphology, yield and its components, disease resistance and vigour using a bi-parental population in chickpea (Varshney et al. 2014a; Roorkiwal et al. 2018a; Sivasakthi et al. 2018). To address the issues related to the narrow genetic diversity and inability of the bi-parental population to deal with multiple traits, multi-parent chickpea populations were developed by combining diverse genetic parent contributions with a high level of recombination (see Varshney et al. 2019b). These multi-parent populations enhance allelic diversity and enable the inclusion of novel recombinants (Huang et al. 2012). For chickpea, nested association mapping (NAM) and multi-parent advanced generation intercross (MAGIC) populations are being developed to create diverse patterns of recombination by making inter-crosses between multiple (4, 8 or 16) parental lines of diverse origin under a balanced funnel crossing scheme to recombined mosaics of founder parents, leading to novel genotype and haplotype combinations. These characteristics are captured for trait mapping, which will increase the genetic diversity in advanced lines.

NAM populations are developed to increase the precision of QTL mapping by combining the strengths of bi-parental mapping populations and association mapping to efficiently capture rare alleles and enhance the chances of identifying novel genomic loci contributing to the trait of interest (McMullen et al. 2009). In chickpea, two MAGIC populations have been developed each at ICRISAT and ICARDA. At ICRISAT, MAGIC population was developed by crossing eight diverse founder parents including cultivars and elite breeding lines (ICC 4958, ICCV 10, JAKI 9218, JG 11, JG 130, JG 16, ICCV 97105 and ICCV 00108) (Gaur et al. 2019). The eight diverse parental lines were crossed in 28 two-way, 14 four-way, and seven eight-way crosses to accumulate recombination events, creating a MAGIC population of 1136 RILs, which were re-sequenced using the WGRS approach. The MAGIC population of ICARDA was developed by crossing of 12 diverse parents to develop a population of 3053 RILs (Hamwieh et al. unpublished data). Analysis of 300 RILs from ICARDA-MAGIC population indicated high diversity of protein ranging from 18 to 31%, and high Fe and Zn contents (> 81 mg Kg^−1^). NAM and MAGIC populations are expected to accelerate the efforts of identification, isolation and transfer of key candidate genes to facilitate chickpea improvement.

## Orphan not any more: advances in genomic resources and technologies

**Fig. 1:**
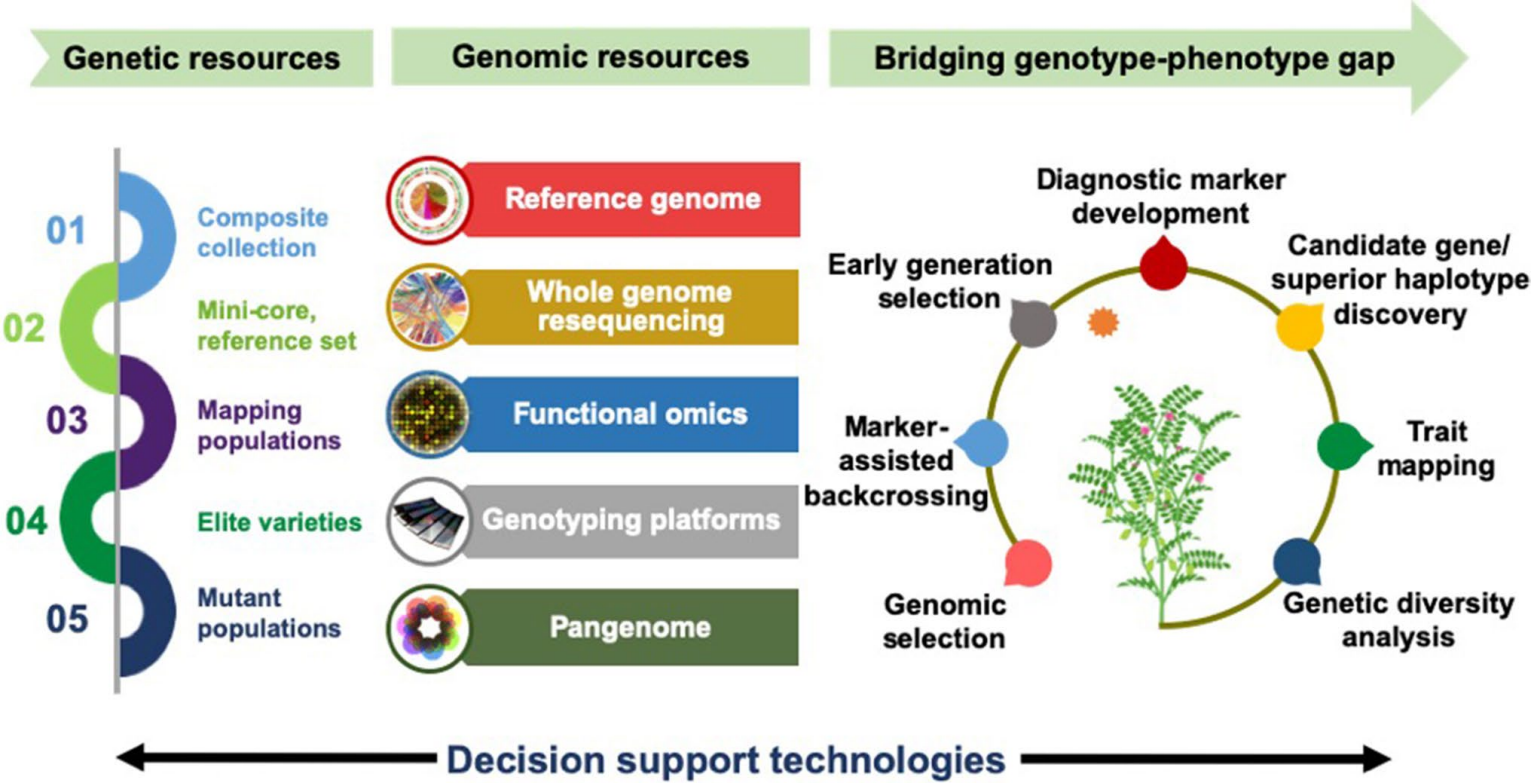
Applications of genomic technologies for bridging the genotype–phenotype gap in chickpea. An overview of genetic resources together with genomic resources and technologies for bridging the genome-to-phenome gap to produce climate-resilient chickpea varieties. Sequencing/genotyping of chickpea germplasm resources, such as composite collection, mini-core and reference set, can be performed. Similarly, elite varieties, mapping populations including bi-parental (recombinant inbred lines, RILs; introgression lines, ILs; F2), multi-parental (multi-parent advanced generation intercrossing, MAGIC; nested association mapping, NAM) populations segregating for important agronomic traits, and mutant populations can also be used. With the availability of the reference genome, these genetic resources can be subjected to whole-genome re-sequencing (WGRS) or high- to low-density genotyping, based on the objective of the study, using the available genotyping platforms (e.g. genotyping by sequencing, GBS; array-based genotyping). Analysis at the transcriptome, proteome and metabolome levels can be performed to gain novel insights into the candidate genes and biological processes involved. A pangenome can be constructed to capture the entire set of genes from *Cicer* species. Analysis of this sequencing/genotyping data along with phenotyping data with high-throughput decision support technologies can provide solutions for genetic diversity analysis, genetic mapping and QTL analysis, identify candidate genes and superior haplotypes, and develop diagnostic marker, early generation selection, marker-assisted backcrossing (MABC) and genomic selection (GS). Integration of such resources should bridge the genotype**–**phenotype gap and accelerate the development of climate change ready varieties with higher yields, improved resistance against biotic and abiotic stresses and enhanced genetic gains in farmers’ fields, particularly in the dryland tropics.

Due to the limited availability of genetic and genomic resources, chickpea was often referred as an orphan crop until 2005. However, with the advancement in NGS technology in the last decade, extensive genetic, genomic and transcriptomic resources have been developed to transform chickpea from an orphan crop to a genomic resource-rich crop (Varshney et al. 2009; Nayak et al. 2010; Hiremath et al. 2012; Thudi et al. 2011; Kudapa et al. 2014; Agarwal et al. 2016; Mashaki et al. 2018). In a similar way, chickpea has graduated from simple pre-curser to one of the principal crops in Ethiopia based on its increasing socio-economic values. The availability of draft genome assemblies, large-scale re-sequencing, molecular markers, low- to high-density genotyping assays and quality check panels have enabled translational genomics in crop breeding (Varshney et al. 2013a, 2019b; Thudi et al. 2016; Rasheed et al. 2017; Roorkiwal et al. 2018a). The development of ultra-high-throughput genotyping platforms that are cost-effective for use in breeding programmes will become a priority for the chickpea community in the coming years. Figure 1 highlights the importance of genomic technologies and resources available in chickpea for bridging the genotype–phenotype gap.

### Whole-genome re-sequencing

NGS technology has resulted in development and application of a wide variety of molecular markers for chickpea improvement (Thudi et al. 2011, 2014; Jaganathan et al. 2015; Kale et al. 2015; Varshney et al. 2018). Over the past decade, more than 2000 simple sequence repeat (SSR) markers, 15,000 features-based diversity array technology (DArT) platform and millions of SNP markers have been developed for chickpea (Varshney 2016). The revolution in NGS technologies has enabled sequencing to be performed at a higher depth (whole-genome re-sequencing), mid-depth (skim sequencing) or lower depth (genotyping by sequencing, RAD-Seq).

Draft genome sequence enables the use of sequencing-based approaches for chickpea improvement. In the first instance, WGRS of parental lines of mapping populations and 100 released cultivars resulted in understanding the genetic diversity among the released lines in addition to numerous variations for high-density trait mapping in chickpea (Thudi et al. 2016). Comprehensive analysis provided a temporal overview of genetic diversity patterns across different time zones (Thudi et al. 2016). In a recent study, the re-sequencing of 429 chickpea accessions from 45 countries identified key candidate genes that were under selection and those associated with agronomically important traits (Varshney et al. 2019a). This study also provided evidences for the origin and migration route of chickpea. Notably, 50,590 high-quality non-redundant SNPs obtained from WGRS data of 429 chickpea lines were used to develop a high-throughput SNP genotyping platform ‘Axiom®*CicerSNP* Array’ (Roorkiwal et al. 2018a), which is now routinely used for trait mapping and QTL detection to advance breeding applications in chickpea. In addition, the recently initiated ‘The 3000 Chickpea Genome Initiative’ is expected to provide novel insights into the underlying genetic variation, domestication patterns and genomic regions associated with important yield component traits in chickpea (Varshney 2016; Varshney et al. 2017). Moreover, extensive use of WGRS data has also provided access to unique alleles, signature sequences and linked markers, among others. In addition, WGRS data on diverse lines allow the development of pangenomes enabling in-depth understanding of species-specific gene(s) and identifying signature genomic regions related to crop domestication and evolution. A recently suggested novel approach called super-pangenome, which includes the development of pangenome of the pangenomes of different species in a given genus, provides an opportunity to identify genus-level genomic variation (Khan et al. 2020). Such advances in technology are expected to unveil significant underlying variations, which will facilitate breeding outcomes in crop species.

### Functional omics approaches

Integrating omics data from multiple platforms such as transcriptomics, proteomics and metabolomics is paramount to bridge the genome-to-phenome gap in crop plants and ultimately identifying the phenotype based on their genetic contribution (Langridge and Fleury 2011; Choi 2019). The information obtained by deploying these types of omics approaches will complement genomic information to manipulate diverse biological processes in chickpea breeding. These omics data are also useful for positional cloning by dissecting the role of genes in the target region linked with an mRNA or protein shift associated with the trait of interest (Strauß et al. 2012; Su et al. 2019).

Recent research efforts in this direction include the deployment of RNA-Seq approach to understand the differential regulation of genes and biological pathways acting under drought stress in kabuli and desi chickpea genotypes. Subjecting BG-362 and P-256 genotypes to polyethylene glycol-stimulated drought stress resulted in the identification of 1,624 differentially expressed genes (DEGs), of which 97 were common for both the genotypes (Kumar et al. 2019a). Furthermore, 4,572 DEGs were identified in shoot and root tissues of kabuli chickpea genotypes (Bivanij and Hashim) under drought conditions, of which drought stress-responsive genes were prioritised (Mashaki et al. 2018). Efforts have also been made to identify DEGs and biological pathways involved in salinity stress conditions. For instance, a total of 3053 DEGs were identified from the root transcriptome of JG 11 (salt-tolerant) and ICCV 2 (salt-sensitive) genotypes subjected to salt stress (Kaashyap et al. 2018). Gaining insight into the patterns of gene expression is crucial for understanding how the underlying genome sequence regulates specific plant phenotypes at key developmental stages of the plant. A comprehensive *Cicer arietinum* Gene Expression Atlas (CaGEA) was developed to cater for this need using RNA-Seq data from different organs and plant developmental stages of drought-tolerant cultivar ICC 4958 (Kudapa et al. 2018).

In addition to transcriptomics, proteomics and metabolomics approaches have been used to identify differentially expressed proteins and metabolites, respectively, under diverse environmental conditions. A comparative analysis of FLIP 97-43C (salt-tolerant) and FLIP 97-196C (salt susceptible) under salt stress identified proteins associated with photosynthesis, stress responsiveness, and protein synthesis and provided novel insights into the salt tolerance mechanism in chickpea (Arefian et al. 2019). Comparative proteomics analysis in JG-62 identified 134 differentially expressed extracellular matrix proteins, including predicted and novel dehydration-responsive proteins involved in a variety of cellular functions (Bhushan et al. 2007). Moreover, a UPLC-HRMS targeted metabolomics approach identified key metabolites that were differentially regulated under drought conditions (Khan et al. 2019). In this case, upregulated metabolites included allantoin, L-proline, L-arginine and L-histidine, and downregulated metabolites included choline, phenylalanine, gamma-aminobutyric acid and alanine. A modern metabolomics strategy based on GC and LC coupled to a triple quadrupole mass spectrometer (GC-QqQ-MS and LC-QqQ-MS) was used to quantitatively profile metabolites in chickpea varieties contrasting for salt tolerance (Dias et al. 2015). While the former approach helped to analyse 48 different metabolites from sugars to organic acids, deploying the latter approach quantified 28 biogenic amino acids expressed under salt stress. That said, studies have also been initiated to integrate functional omics approaches for chickpea genotypes contrasting for drought tolerance, to acquire an overview of the underlying genes and biological mechanisms acting under terminal drought stress. A continuous decrease in the cost of upcoming omics technologies has paved the way for a whole new era of crop molecular breeding.

### Cost-effective genotyping platforms

Vast sequencing information generated using the newly emerging sequencing technologies, has transformed the genomic landscape of crops (Rasheed et al. 2017). These technologies have facilitated the development of marker-based platforms for genotyping in a low- to high-throughput manner. SNPs are considered the marker of choice, due to their wide occurrence, genome-wide distribution, and flexibility for cost-effective and high-throughput genotyping. However, the choice of genotyping platform mainly depends on the objective of the study. For instance, (a) high-density genotyping platforms (20,000 SNPs or more) can be used for genome-wide association study (GWAS), and linkage mapping experiments; (b) medium-density genotyping platforms (2000–10,000 SNPs) can be used for estimating genomic prediction-based genomic selection (GS), genetic diversity analyses and background selection, while (c) low-density genotyping platforms (2–100 SNPs) can be used for gene tagging, marker-assisted backcrossing (MABC), forward breeding and quality control (QC) analysis.

A high-throughput SNP genotyping platform, containing 50,590 SNPs, was developed in chickpea and validated by genotyping two recombinant inbred line (RIL) populations (Roorkiwal et al. 2018a). Notably, the high success and polymorphic rate observed for the genotyping data resulted in developing high-density genetic maps with > 7700 SNPs for each of the RIL populations genotyped. The high-density genotyping platforms are preferred over the medium or low-density genotyping platforms for trait mapping in chickpea, in order to avoid missing data issues that need further imputation for genetic analysis. The sequencing-based genotyping platforms, such as WGRS and skim sequencing, also provide an opportunity for high-density trait mapping (Kale et al. 2015). In addition, mid-density genotyping approaches, such as genotyping by sequencing (GBS) (Jaganathan et al. 2015) and restriction-site-associated sequencing (RAD-Seq) (Deokar et al. 2014), have been successfully exploited for QTL mapping analyses and accomplishing breeding objectives in chickpea. Recent studies suggest that genotyping in a high-density fashion is not mandatory for implementation of GS (see Crossa et al. 2017). Here, alternative multiplexing 2000–5000 informative SNPs are predicted to allow simultaneous marker analyses for increasing MAS efficiency to undertake GS breeding. Therefore, to undertake cost-effective genotyping for deploying GS in chickpea breeding, a 5000 SNP panel is being developed using a targeted sequencing approach (unpublished).

Genotyping using high-density genotyping arrays can be very costly when large breeding populations needed to be genotyped for MAS, backcross breeding, gene tagging and QC analysis. For such applications, few markers are required, and therefore, single-plex high-throughput platforms like KASP, TaqMan and 10-SNP panel are more suitable. For chickpea, cost-effective KASPar assays (Hiremath et al. 2012) and Illumina BeadXpress assays (Roorkiwal et al. 2013) were developed for SNP genotyping for MAS applications. Recent efforts in this direction include the development of 10-SNP panels for carrying out early generation selection in breeding programmes (Varshney et al. 2018). Genotyping with 10 SNP markers can be performed at a cost as low as US$ 1.5–2.0/sample, including DNA isolation, in the high-throughput genotyping project (https://cegsb.icrisat.org/high-throughput-genotyping-project-htpg/) under the ambit of Excellence in Breeding (EiB). This genotyping approach is expected to be extremely useful for remotely located breeding units or those devoid of DNA extraction facilities. The implementation of such small SNP panels for tasks such as early generation screening, seed purity confirmation and identification of true F1s in chickpea breeding programmes is expected to increase in the future.

### Pangenome and super-pangenome

Reference genomes along with whole-genome re-sequencing data have great potential to support crop breeding efforts by providing information about available diversity in the species. However, single genome-based breeding efforts are able to capture limited diversity, and therefore, there is need to deploy pangenome approaches to capture entire diversity present in a species. Scope and applications of pangenome (core and dispensable) have been nicely described by Hurgobin and Edwards (2017). Pangenomes can also differentiate among variations in core (conserved across all individuals of the species) and dispensable (variable among individuals of a species) genomes. Such resources can be of great importance for a crop like chickpea that suffers with narrow genetic base among the cultivated accessions. Breeding efforts in chickpea need to target dispensable genome so that more variations controlling response to various biotic and abiotic stress can be included in chickpea improvement programme. In the case of chickpea, efforts have been initiated to construct the pangenome by sequencing chickpea landraces and varieties. Comparison of the wild species accessions representing from different genepools provides copy number variations and presence–absence mutations that are predicted to be linked with positive selection and agronomically important traits. Therefore, super-pangenome approach has been proposed for developing a pangenome of the pangenomes of different species for a given genus (Khan et al. 2020). In the case of *Cicer* species, de novo genome assemblies are being developed for different *Cicer* species and also a number of accessions representing these species are being re-sequenced for developing super-pangenome. Development of the chickpea pangenome and *Cicer* super-pangenome is estimated to serve as a valuable resource for bridging the genome-to-phenome gap and utilise superior alleles for chickpea improvement.

## Sequencing-based rapid trait mapping

The current genomics landscape of chickpea has provided an opportunity to perform high-resolution genetic mapping. In the post-genomics era, sequencing-based trait mapping is being performed by sequencing the whole population or by sequencing the pooled samples belonging to extreme bulks for the trait of interest (see Varshney et al. 2019b). In this context, low- to high-density sequencing approaches mentioned in the above sections are being used to generate high-density genetic maps and enhance the resolution of trait mapping in chickpea (Table 1).

**Table 1:** An overview of sequencing-based trait mapping efforts in chickpea

S no	Sequencing technology	Mapping population	Trait mapping approach	Target trait(s)	Significant results	Reference
1	GBS	Amit x ICCV 96029	Genetic mapping (3,430 SNPs)	Ascochyta blight resistance	QTLs for resistance on CaLG02, CaLG03, CaLG04, CaLG05 and CaLG06 explaining up to 40% of phenotypic variance (PVE)	Deokar et al. (2019a)
2	GBS	ICC 4567 × ICC 15614	Genetic mapping (271 SNPs)	Heat tolerance	QTLs on CaLG05 and CaLG06 with a cumulative PVE of 51.89% and 25.84%, respectively	Paul et al. (2018)
3	GBS	SBD377 x BGD112	Genetic mapping (3,228 SNPs)	Seed trait	QTLs explaining up to 29.71% PVE and candidate genes for seed traits	Verma et al. (2015)
4	GBS	ICC 4958 × ICC 1882	Genetic mapping (743 SNPs)	Drought tolerance-related traits	Refined the *"QTL-hotspot"* region from ca. 29 cM to 14 cM and added 49 SNPs in the region	Jaganathan et al. (2015)
5	GBS	Cultivated and wild accessions	GWAS, genetic mapping	Photosynthetic efficiency and seed yield per plant	SNPs and candidate genes associated with photosynthetic efficiency and seed yield per plant	Basu et al. (2019)
6	GBS	Cultivated accessions	Genetic diversity (4,349 SNPs)	Seed yield per plant	A pentricopeptide repeat (PPR) gene associated with seed yield per plant	Basu et al. (2018)
7	GBS	Cultivated accessions	GWAS (16,591 SNPs)	Seed iron and zinc	Genomic loci/ genes (with 29% combined PVE) associated with seed-Fe and Zn concentrations	Upadhyaya et al. (2016)
8	GBS	Cultivated and wild accessions	Genetic diversity (82,489 SNPs)	NIL	Analysis of genetic diversity, population structure and linkage disequilibrium	Bajaj et al. (2015)
9	GBS	Cultivated and wild accessions	Genetic diversity (44,844 SNPs)	NIL	Revealed complex admixed domestication pattern, and extended LD decay	Kujur et al. (2015)
10	GBS	Cultivated accessions	Genetic diversity (3,187 SNPs)	NIL	Genetic cluster associated with black seeded genotypes	Pavan et al. (2017)
11	RAD-Seq	ICCV 96029 × CDC Frontier	Genetic mapping (604 bins)	NIL	High-density linkage map constructed	Deokar et al. (2014)
12	Axiom Array	ICC 4958 × ICC 1882 & ICC 283 × ICC 8261	Genetic mapping (13,679 and 7,769 SNPs)	Drought tolerance-related traits	Main-effect QTLs for several drought component traits	Roorkiwal et al. (2018b)
13	WGRS	ICC 4958 × ICC 1882	Genetic mapping	Drought tolerance-related traits	Delimited *"QTL-hotspot"* region to ~ 300 kb and identified 26 candidate genes	Kale et al. (2015)
14	WGRS	ICC 4958 × ICC 1882	Genetic mapping	Plant vigour and canopy conductance	QTLs for plant vigour and canopy conductance traits on CaLG04 and CaLG03, respectively	Sivasakthi et al. (2018)
15	WGRS	ICCV 96029 × CDC Frontier and ICCV 96029 × Amit	QTL-seq	Ascochyta blight resistance	Candidate genes for ascochyta blight resistance	Deokar et al. (2019b)
16	WGRS	ICC 4958 × ICC 1882	QTL-seq	100-seed weight (100SDW) and root/total plant dry weight ratio (RTR)	Genomic regions on CaLG01 (1.08 Mb) and CaLG04 (2.7 Mb) linked with 100-seed weight. Two genes (Ca_04364 and Ca_04607) for 100SDW and one gene (Ca_04586) for RTR	Singh et al. (2016)
17	WGRS	ICC 7184 × ICC 15061	QTL-seq	100-seed weight	Genomic region on CaLG01 harbouring six candidate genes for 100-seed weight	Das et al. (2015)
18	WGRS	Released varieties and advanced breeding lines	GWAS (144,000 SNPs)	Drought tolerance	MTAs significantly associated with yield and yield-related traits under drought	Li et al. (2018)

*GBS* Genotyping by sequencing, *RAD-Seq *restriction-site-associated sequencing, *WGRS* whole-genome re-sequencing

In recent years, the GBS approach has been used to detect genome-wide SNPs in chickpea to understand allelic diversity and population structure, and develop high-density linkage maps, QTL analysis, GWAS and GS. For example, the GBS approach has been widely used for linkage mapping and QTL detection of ascochyta blight resistance (Deokar et al. 2019a), heat tolerance (Paul et al. 2018), seed iron and zinc concentrations (Upadhyaya et al. 2016) and seed quality (Verma et al. 2015) among others, using RIL populations in chickpea (Table 1). This technology has also been used to identify and validate SNPs from cultivated and wild *Cicer* accessions to study allelic diversity, population structure and linkage disequilibrium patterns in these gene pools (Bajaj et al. 2015; Kujur et al. 2015; Pavan et al. 2017). Interestingly, the GBS approach was used for enhancing marker density within the ‘*QTL-hotspot*’ region, which harbours multiple QTLs for drought tolerance. This approach successfully delimited the locus from ~ 29 to ~ 14 cM on the genetic map (Jaganathan et al. 2015). Recently, GBS was used for high-resolution association analysis and GWAS integrated with QTL mapping and transcript profiling in germplasm lines and mapping populations to detect molecular signatures regulating photosynthetic efficiency in chickpea (Basu et al. 2019). The RAD-Seq technology was used to construct a high-density genetic map using an intraspecific population and provided novel insights into the frequency of recombination and hot-spots across the chickpea genome (Deokar et al. 2014). Although WGRS and GBS have been widely used for trait mapping and QTL detection in chickpea, both approaches have their own limitations; GBS generates numerous missing data points across the genome, and WGRS is costly for sequencing large mapping populations. These limitations can be partly offset by sequencing at low coverage, referred to as skim sequencing (Golicz et al. 2015). This approach was used to fine map the ‘*QTL-hotspot*’ region, from ~ 3 Mb to ~ 300 kb, enabling identification of key genes related to drought tolerance in chickpea (Kale et al. 2015).

Recent trait mapping approaches (QTL-seq, Indel-seq, Seq-BSA, MutMap and BSR-seq) mainly rely on sequencing bulk samples of RILs displaying extreme phenotypes specific to the trait under study (see Varshney 2016). Of these, ‘QTL-seq’ has been successfully used for mapping trait of interest in chickpea (Das et al. 2015; Singh et al. 2016; Deokar et al. 2019b). The first QTL-seq study in chickpea used lines with extreme phenotype from an intraspecific mapping population (ICC 7184 × ICC 15061) to identify the candidate gene responsible for 100-seed weight (Das et al. 2015). Interestingly, this study identified a coding SNP (G/A) in the constitutive photomorphogenic9 (COP9) signalosome complex subunit 8 (CSN8) gene, which displayed a close association with the major 100-seed weight robust QTL (CaqSW1.1). The evolutionarily conserved multi-protein gene complex present within CSN8 gene is predicted to be involved in controlling growth and development by modulating multiple E3 ubiquitin ligases and auxin-response pathways in crops (Serino and Deng 2003; Schwechheimer and Isono 2010). In addition, a QTL-seq analysis performed in a ICC 4958 × ICC 1882 RIL population identified two candidate genes (Ca_04364 and Ca_04607) for 100-seed weight and one candidate gene (Ca_04586) for root dry weight/total plant dry weight ratio (RTR) in chickpea (Singh et al. 2016). For 100-seed weight, the gene Ca_04364 encoded a ‘cell division protein kinase’ while Ca_04607 encoded a ‘transmembrane protein’ predicted to regulate grain characteristics in rice (Fan et al. 2006; Shomura et al. 2008). Moreover, Ca_04586 gene coding for ‘cytochrome P450 monooxygenase’ has been previously reported to be involved in regulating root development and drought tolerance in tobacco (Duan et al. 2017) and maintenance of ABA levels in crop plants (Kushiro et al. 2004). In a recent study, an NGS-based bulked segregant analysis (BSA) approach was used to identify 11 QTLs and 6 QTLs in two RIL populations CPR-01 and CPR-02, respectively, for ascochyta blight resistance in chickpea (Deokar et al. 2019b). The genomic regions identified in these studies helped to identify potential candidate genes and facilitate the development of diagnostic markers for pyramiding numerous QTLs for agronomically important traits in chickpea.

## Marker-assisted backcrossing for the development of superior varieties

Integrating molecular technologies in crop breeding programmes can enhance yield, nutrition and pest/disease resistance while simultaneously addressing the challenges of climate change. Collaborative efforts among multiple organisations have implemented genomic technologies in crop breeding programmes to deliver several molecular breeding products for chickpea in recent times. Among the genomics-assisted breeding (GAB) approaches used for crop improvement, including MABC, marker-assisted recurrent selection (MARS) and GS, the MABC/MAS approach delivered improved chickpea varieties with higher yield and climate resilience in recent years. Details pertaining to the recently released chickpea varieties using molecular breeding approaches are provided below and summarised in Table 2.

**Table 2:** List of molecular breeding products developed using marker-assisted breeding (MABC) in chickpea

S no	Trait	Donor parent	Recipient parent	Target region	Present status/ released variety	Reference
1	Drought tolerance	ICC 4958	JG 11	*QTL-hotspot*	Released as 'Geletu' for commercial cultivation in Ethiopia	EIAR/ICRISAT unpublished
2			Pusa 372	*QTL-hotspot*	Released as 'Pusa chickpea 10216′ for commercial cultivation in India	ICAR-IARI/ICRISAT unpublished
3			JG 11	*QTL-hotspot*	Stable backcross lines under multi-location trials	Varshney et al. (2013b)
4			RSG 888	*QTL-hotspot*	Under multi-location yield trials of ICAR-AICRP for release in India	Unpublished^**#**^
5			Pusa 362	*QTL-hotspot*	Under multi-location yield trials of ICAR-AICRP for release in India	Unpublished^**#**^
6			JAKI 9218	*QTL-hotspot*	Under multi-location yield trials of ICAR-AICRP for release in India	Unpublished^**#**^
7			DCP 92-3	*QTL-hotspot*	Under multi-location yield trials of ICAR-AICRP for release in India	Unpublished^**#**^
8			JG 11	*QTL-hotspot*	Under multi-location yield trials of ICAR-AICRP for release in India	Unpublished^**#**^
9			ICCV 10	*QTL-hotspot*	Under multi-location yield trials of ICAR-AICRP for release in India	Unpublished^**#**^
10	Fusarium wilt resistance	WR 315	Annigeri 1	*foc4*	Released as 'Super Annigeri 1′ for commercial cultivation in India	Mannur et al. (2019)
11			JG 74	*foc4*	Under multi-location yield trials of ICAR-AICRP for release in India	Mannur et al. (2019)
12			Pusa 372	*foc4*	Under multi-location yield trials of ICAR-AICRP for release in India	Unpublished^**#**^
13			Pusa 391	*foc4*	Under multi-location yield trials of ICAR-AICRP for release in India	Unpublished^**#**^
14		Vijay	Pusa 256	*foc 2*	Under multi-location yield trials of ICAR-AICRP for release in India	Pratap et al. (2017)

^#^*Source*—Annual Report 2018-19-All India Coordinated Research Project on Chickpea

### Geletu, a drought-tolerant variety released in Ethiopia

The MABC approach was successfully implemented at ICRISAT by introgressing the ‘*QTL-hotspot*’ from ICC 4958 in the popular Indian chickpea cultivar JG 11. The ‘*QTL-hotspot*’ region is a promising target region in molecular breeding for developing drought-tolerant chickpea varieties (Varshney et al. 2013b). Here, the ‘*QTL-hotspot*’ region from ICC 4958 was introgressed in JG 11 using MABC. Subsequently, in Ethiopia, a multi-location evaluation of the MABC lines facilitated the release of a high-yielding chickpea variety, locally named as ‘Geletu’—means thanks God—with pedigree [(JG 11 × ICC 4958) × 3*JG 11] – 29], which was named after the eminent pulse scientist late Dr Geletu Bejiga from EIAR, Ethiopia. This variety was officially released for commercial cultivation in Ethiopia by the National Variety Release Committee (NRVC), Ethiopia, and was commended for wider adoption in the dryland tropics to moist agro-ecological zone ranges. Notably, the variety had the highest grain yield of 3822 kg/ha at Arsi Robe, which corresponded to a yield advantage of about 15% over the check variety ‘Teketay’. Geletu also produced 100-seed weight ranging from 28 to 39.9 g as compared to recurrent parent at different locations. In Ethiopia, Geletu showed resistance against Fusarium wilt and root rot disease with disease ratings of 3 and 2, respectively, on a scale of 1–9 (where 1 = disease resistant, 9 = disease susceptible). Ethiopia became the first country to release a climate-resilient chickpea variety developed using marker-assisted backcrossing efforts.

### *Pusa chickpea 10216*, a drought-tolerant variety released in India

Pusa 372, a popular chickpea variety developed by ICAR-Indian Agricultural Research Institute (IARI) in 1993, was grown in the central zone, north-east and north-west plains zones of India and used as a control variety in late-sown national chickpea trials. However, in recent years, the productivity of this variety declined under drought conditions. Therefore, ICAR-IARI, in collaboration with ICRISAT, initiated efforts to enhance drought tolerance of ‘Pusa 372′ by introgressing the ‘*QTL-hotspot*’ region from ICC 4958 using the MABC approach. Based on yield performance in multi-location trials across India in the ICAR–All India Coordinated Research Project (AICRP) on chickpea, an improved Pusa 372 was released under the name ‘Pusa chickpea 10216’ for commercial cultivation in India. ‘Pusa chickpea 10216’ variety had a 11% yield advantage over its recipient parent under moisture stress in the central zone of India and a substantial increase in 100-seed weight. Notably, ‘Pusa chickpea 10216’ is the first molecular breeding variety with enhanced drought tolerance released in India.

### Super Annigeri 1 and improved JG74 lines resistant to Fusarium wilt in India

The MABC approach has also been used to improve Fusarium wilt (FW) resistance in two elite high-yielding desi cultivars, Annigeri 1 and JG 74, at the University of Agricultural Sciences, Raichur (UAS-Raichur) and Jawaharlal Nehru Krishi Vishwa Vidyalaya (JNKVV), Jabalpur, India, respectively, in collaboration with ICRISAT. In recent years, both cultivars showed high susceptibility to FW race 4 (foc4), an important factor reducing yield in Central and South India. Therefore, to improve their resistance, a genomic region conferring resistance against foc4 was introgressed using MABC, with WR 315 as the donor parent. Evaluation of backcross lines at various locations (wilt sick plots) throughout India demonstrated a yield advantage of about 8% and enhanced disease resistance over the recipient parent, Annigeri 1 and was released as ‘Super Annigeri 1’ for commercial cultivation in India (Mannur et al. 2019). Due to its potential to overcome FW, this variety was released in different states of India, including Andhra Pradesh, Karnataka, Maharashtra and Gujarat by ICAR-AICRP on chickpea. Furthermore, a multi-location evaluation of backcross lines in the JG 74 background identified a superior line, JG 74315–14, with 25.6% to 53.5% increase in yield at Durgapura and Pantnagar areas, respectively, in the initial varietal trial of ICAR-AICRP on chickpea (Mannur et al. 2019).

In addition to the above-mentioned released chickpea varieties, several molecular breeding lines, developed using the MABC approach for drought tolerance and FW resistance in diverse background (Varshney et al. 2014b; Pratap et al. 2017), are being assessed in multi-location trials by ICAR-AICRP on chickpea. These lines with high-yield performance and enhanced resistance to biotic and abiotic stresses demonstrate the potential of integrating genomics with breeding efforts to develop superior climate-resilient varieties in chickpea.

## Genomic selection for selecting loci with relatively small genetic effects

A significant limitation of marker-assisted selection is that only major QTLs/genes can be targeted. It is now well known that many of the complex traits involving yield or broad-spectrum disease resistance consist of multiple genomic regions, each with relatively small genetic effects. In many cases, it is highly desirable to select for all or multiple QTLs associated with the trait of interest. In this context, genomic selection which has the potential to capture several genes with minor additive effects can be useful in crop breeding. Genomic selection has been broadly used in animal breeding programmes and its popularity for enhancing genetic gains in crop breeding is on the rise. A rapid increase in cost-effective sequencing technologies and high-throughput phenotyping facilities is providing new avenues for implementation of GS to improve gains in crop plants. Analysis of the genotypic and phenotypic data set of a ‘training population’ enables the estimation of genomic estimated breeding values (GEBVs) that are used for subsequent selections under GS (Meuwissen et al. 2001). This approach is practical for increasing selection efficiency in crops by reducing the length of breeding cycles, eliminating the phenotyping required for successive selection, thereby reducing the time and cost, and leading to higher genetic gains. Genomic prediction which depends mainly on the availability of high-throughput genotyping in addition to accurate phenotyping data is the key to success in GS breeding.

The availability of large amount of genomic resources and high-density genotyping data has facilitated the successful implementation of GS in chickpea (Roorkiwal et al. 2016, 2018b; Li et al. 2018). Phenotyping and genotyping data collected from 320 elite breeding lines were combined with six statistical GS models to evaluate prediction accuracies. Prominent results were evident from the high prediction accuracies (up to 0.91) obtained for diverse yield and its component traits for GS in chickpea breeding (Roorkiwal et al. 2016). A recent GS study suggested the possibility of increasing prediction accuracies for complex traits like drought by incorporating GWAS results into GS models (Li et al. 2018). This study also provided evidence that the application of GS models using a subset of SNPs closely associated with the trait, in contrast to all SNPs, can increase prediction accuracies by several folds for yield-related traits. Application of GS in crops is hindered by several factors such as limited size of training population, similarity between training population and testing population, number markers, and the integration of genotype × environment (G × E) and marker × environment (M × E) interactions, to name a few (see Crossa et al. 2017). The inclusion of G × E effects in GS models can improve the prediction accuracies in breeding programmes (Roorkiwal et al. 2018b). This study also highlighted the estimation of higher prediction accuracies using DArT Seq when compared with GBS. Several approaches including GS + de novo GWAS and haplotype-based GS + de novo GWAS have potential for developing promising chickpea genotypes.

## Speed breeding for fast-forwarding genetic gains

The current pace of varietal development and genetic gains will not meet future food demands, particularly with the burgeoning population and climate change scenarios (Hickey et al. 2019). It takes several years for an improved variety to be developed and released due to prolonged breeding cycles. Genetic gains (∆*G* = (σ*a*)(*i*)(*r*)/*L*) generally regarded as the ‘breeder’s equation’ can be enhanced by shortening the breeding cycles (*L*); this rapid generation advancement can be attained by modulating temperature, humidity, photoperiod and harvesting/germination of immature seeds (Watson et al. 2018). In this scenario, recently developed ‘speed breeding’ technology in chickpea will be advantageous for increasing productivity gains and shortening life cycles that will allow researchers to undertake more generations per year (Watson et al. 2018; Hickey et al. 2019).

Most breeding programmes use selection–recombination–selection cycle to develop new varieties, with production requiring 5–6 generations to reach the genetic homozygosity before testing for their performance and stability. However, challenges such as crop duration, photoperiod and temperature sensitivity hinder multiple generations per year. Double haploids (Ren et al. 2017), in vitro culturing of immature embryos (Wang et al. 2009), embryo rescue technique (Rizal et al. 2014), simplified biotron technique (Tanaka et al. 2016) and others have been used for rapid generation cycling in many crops, but no such successes have occurred in chickpea. The first report on rapid generation advancement in chickpea provided evidence for three generations per year in short-season environments (Gaur et al. 2007). Recently, a speed breeding protocol for chickpea was developed with up to seven generation cycles per year. The study undertook two generations in the field during winter and spring seasons and the third generation in pots during the rainy season by making use of a cost-effective rainout shelter to protect crops from excess rains. Extended photoperiod under glasshouse conditions led to early flowering and seed germination, which reduced the generation cycle time (Samineni et al. 2019). Moreover, recent studies have predicted the possibility of producing 4–6 generations per year in chickpea under speed breeding conditions with extended photoperiod and controlled temperature regime (Watson et al. 2018; Ghosh et al. 2018).

The integration of novel technologies into crop breeding programmes could be a significant leap for improving agricultural productivity (Hickey et al. 2019). This technology will help to: (1) accelerate the development of mapping populations, such as RILs, NILs, MAGIC and NAM for trait mapping, (2) develop improved varieties faster by advancing MABC/MAS generation cycles and (3) fast-track genebank mining and enhance genetic gains by integrating with GS and GAB, respectively.

## Sequence-based holistic breeding approach

Genomics research in early phase focused on model species, predominantly *Arabidopsis thaliana*, for comprehensive information on genes, their regulatory functions and the performance of their products. At the moment, genomics is at a fascinating juncture for crop plants that has provided novel insights on the role of plant responses to ecological stress, diseases and pests, and it is limited in its ability to provide practical applications for crop improvement. Rapid advances in genomics technologies have made it possible to generate extensive data sets for several crop plants, enabling breeders to tackle problems related to agronomic crop performance that were difficult to address previously.

**Fig. 2:**
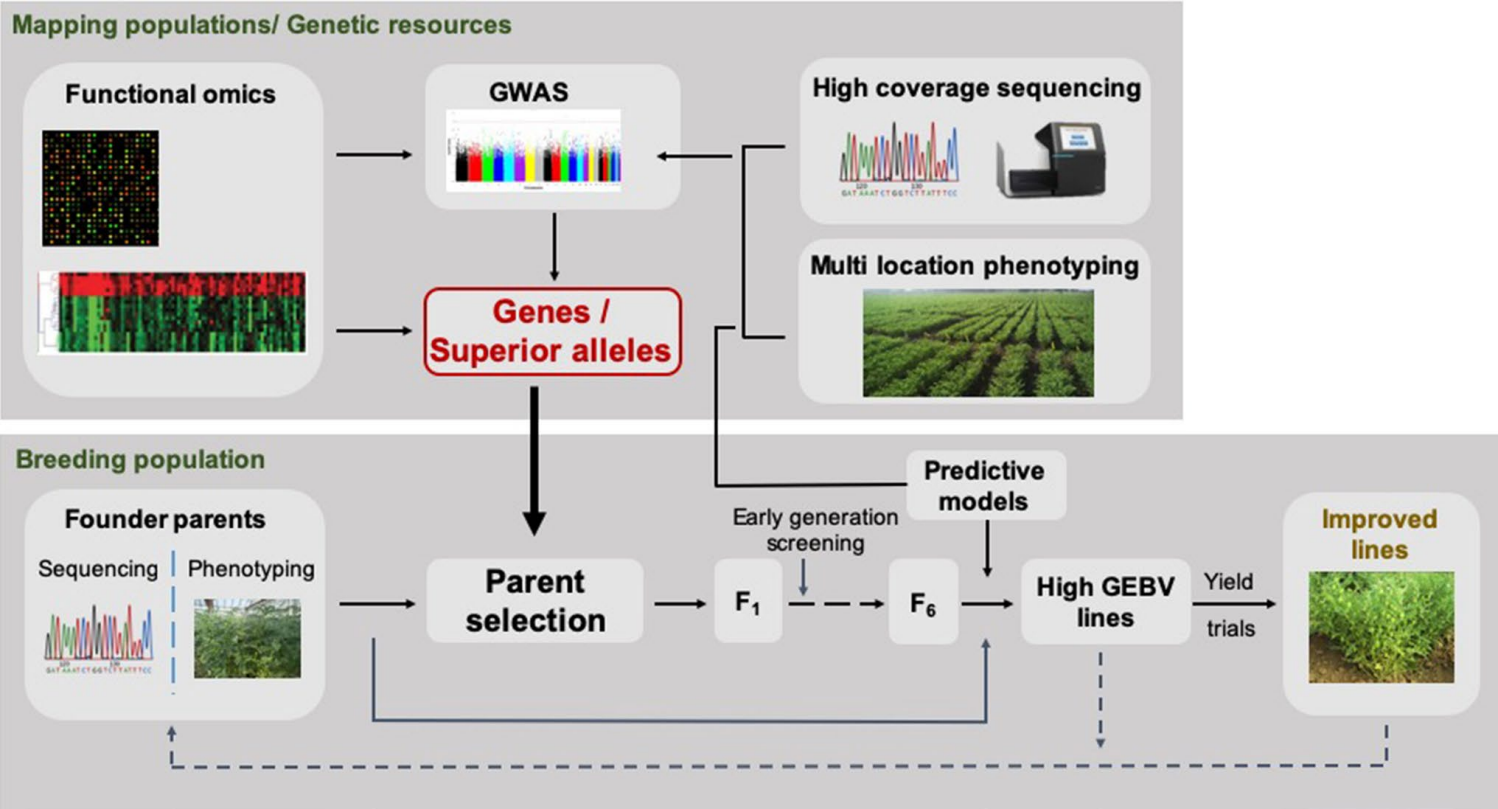
Sequence-based holistic breeding approach to accelerate genetic gains in chickpea breeding. Integration of functional omics, GWAS, parent selection, high-throughput sequencing and phenotyping, and genomic selection to improve and accelerate the development of superior chickpea varieties in breeding. Multi-omics data can enable positional cloning by providing information on genes/superior alleles in the target region. High-throughput sequencing and multi-location phenotyping will harness superior alleles with diverse genetic resources. Newly identified genes/superior alleles can feed directly into the breeding population for parental selection. Models can also be used to predict the genomic estimated breeding values (GEBVs) based on high-coverage sequencing and phenotyping data. The integration of sequencing-based approaches with breeding programmes will be reflected in high productivity gains under limited resources and time

Several molecular breeding approaches including MABC, MAS and forward breeding are being used to introgress genomic loci into elite and leading crop varieties including chickpea (Varshney et al. 2018). While these approaches have been successful, they are limited in their ability to introgress only three gene combinations at a given time. Stacking numerous genes in a single genetic background through backcrossing or assembling them through forward breeding approaches is challenging. One possible solution will be to integrate sequence-based approaches into breeding programmes that will help to fill the remaining gaps in the relationship between genotype and phenotype. In this context, we provide a sequence-based holistic breeding approach that can be used in chickpea breeding programmes (Fig. 2). This approach involves sequencing and phenotyping all possible parental lines from a given mapping/breeding population and/or germplasm lines in a high-throughput fashion. Transcriptomics, proteomics and metabolomics levels can also be assessed to understand the underlying biological processes at multiple levels. Approaches like GWAS can be performed to integrate the sequencing and phenotyping data to identify genes/superior alleles linked with the trait of interest. These data will enable selection of suitable parent combinations with a high frequency of superior alleles and low frequency of deleterious alleles in the mapping population. The selected parental lines can be crossed with each other to obtain F_1_s, and early generation screening of the progenies can be performed using the available 10 SNP panels for chickpea. A set of germplasm lines can be used to develop the training model for performing GS analysis on select lines from the breeding population.

Further, modern technologies, such as speed breeding, can be used to fast-forward generation cycles of this population. The progenies of the breeding population can be sequenced at a lower depth using skim-sequencing approaches in the F_6_ generation. Consequently, superior lines containing higher GEBVs can be selected for subsequent yield trials at multiple locations, leading to development of improved varieties. Improved lines can be re-integrated into crossing programmes to increase the efficiency of the next round of breeding populations. We are hopeful that a sequence-based holistic breeding approach for chickpea will lead to the continuous development of breeding populations and increase the rate of genetic gains at the end of each breeding cycle.

## Way forward

Innovations in NGS technologies integrated with high-throughput phenotyping have enabled genomics to be applied in diverse streams of crop breeding. But can genomics-assisted breeding improve chickpea varieties to meet the nutritional demands of the people from the dryland tropics? The development of high-throughput genotyping platforms for use in applied breeding programmes is on the rise. However, are there any alternative multiplex or array-based approaches available for medium-density genotyping needs in chickpea breeding? Can we develop an integrated chip containing diverse genomic information for genome-wide screening in chickpea?

Micronutrient malnutrition is becoming a significant concern in developing countries in Asia and sub-Saharan Africa where several million children and pregnant women are affected (Kumar et al. 2019b). In this context, it is important to develop biofortified chickpea varieties that can address micronutrient deficiencies. A significant limitation preventing conventional biofortification approaches in achieving success is that the uptake and accumulation of micronutrients in edible parts of the crops are controlled by multiple genes with relatively small effects (Naqvi et al. 2009). Moreover, the success achieved using this approach is influenced by the natural allelic diversity present in the gene pool. To this end, a GS approach that can capture superior alleles with minor effects would be ideal for developing improved chickpea varieties that are rich in micronutrients. The market and farmers’ preferred traits should form the base of breeding programmes so that consumers’ demand can be fulfilled. The future market will favour cultivation of extra-short duration varieties having immature green grains of desi chickpea for their use as vegetables, matured green seeded chickpea and small seeded chickpea for sprouts. The market value of chickpea is mainly decided by colour, shape and size of the grain. In recent years, there has been demand for large seeded (> 25 g/100 seeds) desi chickpea of 9–10 mm seed size mostly for parching. Nowadays, most kabuli breeding programmes have shifted emphasis to lager seed size for getting high premiums. Several studies have indicated the role of soil microbiota in managing soil pathogens in suppressive soils. Differences have been observed among genotypes of chickpea for their ability to alter soil microbiome, which opens another dimension for selecting chickpea genotypes enabling beneficial soil biotic environment (Ellouze et al. 2013). This needs to be explored further to select cultivars that improve rhizosphere health leading to efficient use of soil resources. Considering increasing labour wages, erect and tall growth habit is becoming most preferred traits for chickpea due to their amenability to machine harvesting (Chaturvedi et al. 2014). Erect plant types also ensure better sunlight penetration inside crop canopy leading to reduced humidity and in turn less incidence of foliar diseases. In addition, chickpea community has also started to focus on post-harvest operations along with herbicide tolerance to avoid time-consuming and expensive harvest and weed control (Gupta et al. 2018; Dixit et al. 2019).

Although the revolution in genotyping technologies has provided a range of platforms for gene discovery and molecular breeding in crops, the choice of genotyping approach depends on the nature of the study. High-density chip-based platforms for applications like GWAS and linkage mapping are now widely used in chickpea. While applications such as gene tagging, backcross breeding and marker-assisted recurrent selection are being achieved using single-plex genotyping platforms such as KASP and TaqMan, these technologies would be very costly and time-consuming for GS and diversity analysis. In this case, a multiplex assay that will analyse multiple markers simultaneously could be developed to increase the efficiency of MAS and GS breeding in chickpea. Here, a sequence-based platform containing 2000–5000 SNPs could be developed for multiplex medium-density SNP genotyping in chickpea. Having achieved this, there is a dire need to develop an integrated chip that can accommodate genomics information at diverse levels, such as SNPs, CNVs, InDels and epigenetics, in chickpea. It is anticipated that these resources will be routinely used for chickpea improvement in the coming years.

## Summary

Crop improvement depends mainly on the underlying genetic variations. Incremental genetic gains can be achieved by either enhancing the amount of genetic variation available for selection or increasing the speed, efficiency and accuracy of the selection to develop varieties rapidly having market-oriented traits. Genomics technologies have the potential to support both approaches. Optimum genomic resources are now available for chickpea, which can be used in breeding programmes. We hope that MAS/MABC approaches continue to improve elite/leading chickpea varieties for select traits via molecular breeding and that parent selection, screening early generation and GS are integrated, either in combination or individually, into chickpea breeding efforts to accelerate the rate of genetic gains. Although genotyping technologies will be more affordable in the coming years to facilitate sequencing of the entire chickpea population, high-quality phenotyping data for these lines will be needed to enable trait discovery. Several other resources including mathematical models, data analysis skills, field experiment design, barcode labelling and databases for storing genotyping and phenotyping data will be important for the successful execution of breeding efforts. To this end, multi-institutional collaborative initiatives, such as the EiB platform (https://excellenceinbreeding.org/) and Integrated Breeding platform (https://www.integratedbreeding.net/), will play a crucial role in addressing the challenges faced in chickpea breeding.

Integrating a sequence-based holistic breeding approach for chickpea improvement has the potential to develop superior varieties and provide substantial productivity gains. Here, the use of functional genomics approaches and genome-wide selection will help to capture genes and superior alleles, respectively, for inclusion in molecular breeding programmes to bridge the gap between genotype and phenotype. In conclusion, we see enormous potential of genomics technologies to modernise breeding approaches to deliver next-generation chickpea varieties, which will have a substantial impact in farmers’ fields.
